# Dissimilar local risk factors among patients diagnosed with cystic echinococcosis upon voluntary screening in highly endemic regions of Kyrgyz Republic

**DOI:** 10.1017/S0031182024000763

**Published:** 2024-09

**Authors:** Kursanbek M. Raimkulov, Vera S. Toigombaeva, Omurbek T. Kuttubaev, Zhyldyz S. Smailbekova, Asel D. Adambekova, Maria N. Ruzina, Lyudmila V. Akhmadishina, Alexander N. Lukashev

**Affiliations:** 1 Kyrgyz State Medical Academy named after I.K. Akhunbaev, Bishkek, Kyrgyz Republic; 2 Martsinovsky Institute of Medical Parasitology, Tropical and Vector Borne Diseases, Sechenov University, Moscow, Russia

**Keywords:** cystic echinococcosis, *Echinococcus*, endemic, prevalence, risk factors, transmission

## Abstract

Echinococcosis is a parasitic invasion caused by a cestode of the genus *Echinococcus*. Kyrgyzstan is a country in Central Asia known for an extremely high incidence of echinococcosis. A total of 10 093 subjects were screened in the Osh, Naryn and Batken regions of Kyrgyzstan in 2015–2017 by ultrasound and questioned for potential risk factors. Cystic echinococcosis (CE) prevalence (combined newly diagnosed and post-surgery cases) ranged between 0.2 and 25.2% across the study regions. Typical factors, such as dog or livestock ownership, weakly affected CE risk (odds ratio [OR] = 1.18–1.83). Use of water from a well and owning a cat had a greater effect on CE risk (OR = 2.02–2.28). The risk factors of CE were highly dissimilar among the study regions, with patterns not always compatible with classical biohelminthosis transmission routes (no risk from livestock in certain areas, significant risk from using well water, owning cats). Therefore, the CE epidemic in Kyrgyzstan is not holistic in terms of potential mechanisms and risk factors, and certain areas can greatly benefit from preventive measures that will have limited efficiency elsewhere.

## Graphical abstract

**Figure:**
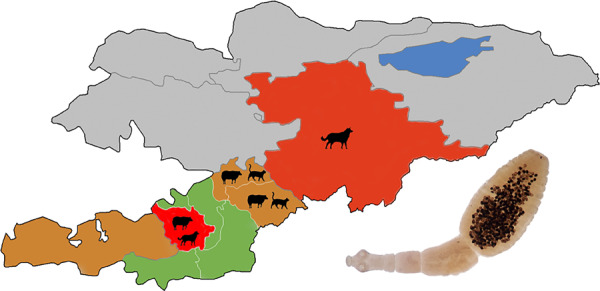

## Introduction

Echinococcosis is a parasitic invasion caused by a cestode of the genus *Echinococcus*. In humans, the disease is caused by the larval form, and there are 2 principal varieties, alveolar echinococcosis (AE), caused by *Echinococcus multilocularis*, and cystic echinococcosis (CE) caused by *Echinococcus granulosus*. The life cycle consists of a mature parasite that lives in the intestine of carnivores, and a larval (metacestode) stage that develops mainly in herbivorous animals (CE) or rodents (AE) (Eckert and Deplazes, 2004). AE features malignant growth in humans and may be relatively resistant to cure, whereas CE is more benign. It manifests as cysts (predominantly located in the liver, less frequently in the lungs, rarely in other organs or even in the muscle tissue) and often develops undiagnosed for many years. The most common and socially relevant variant of the CE invasion cycle consists of livestock and dogs, although a wide range of alternative ecological cycles and infection sources are possible (Eckert and Deplazes, 2004).

Kyrgyzstan is a country in Central Asia that is known for an extremely high incidence of both CE and AE (Torgerson, 2013; Usubalieva *et al*., 2013). One recent report indicated surgical incidence of 13.1 per 100 000 population per year across the whole country, and up to 176 per 100 000 per year in endemic districts (Paternoster *et al*., 2020). The epidemic of echinococcosis in Kyrgyzstan is a part of a general epidemic in Central Asia that began after the collapse of the Soviet Union (Torgerson, 2013) and was attributed to changing agricultural practices: closure of large organized collective farms that could afford adequate sanitation, on the one hand, and cessation of nomad-style sheep breeding, which separated animal husbandry from farming, on the other (Shaikenov *et al*., 2003). The prevalence of CE (surgical/ultrasound) in humans increased from 2.8% in 1987 to 8.2% in 1998 (Raimkulov, 2020), and the incidence peaked in 2014 at 20.2 per year per 100 000 population. Major control efforts have been implemented after 2008, and a slow decline in CE incidence has been observed after 2016 (Raimkulov, 2020). The country has variable landscape and human lifestyle patterns, resulting in highly dissimilar echinococcosis incidence in specific regions (Paternoster *et al*., 2020).

Before 2014, CE was diagnosed in Kyrgyzstan almost exclusively upon surgical case reporting. In 2014, a large ultrasound and enzyme-linked immunosorbent assay (ELISA) screening study was implemented in south Kyrgyzstan. This work analyses risk factors that have been associated with CE prevalence in highly endemic regions of south and central Kyrgyzstan among patients diagnosed with CE upon screening.

## Materials and methods

### Dataset

**Figure 1.**
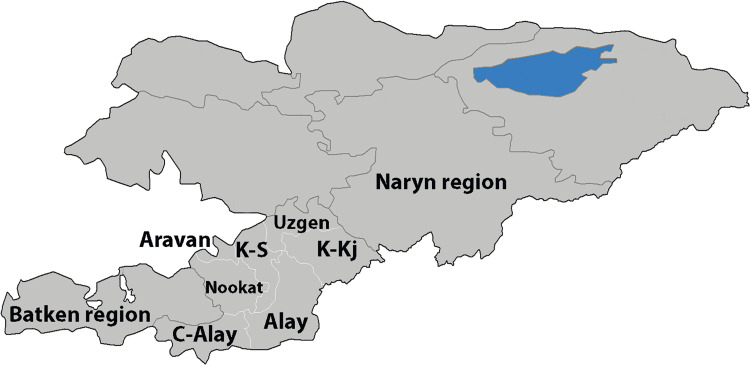
Map of the regions (oblast’) and districts (raion) of the Osh region of Kyrgyzstan covered in the study. C-Alay, Chon-Alay; K-Kj, Kara-Kulja; K-S, Kara-Suu. Modified from the original image available at Wikimedia Commons, Created by Rarelibra 3 August 2007.

This study uses a dataset that was assembled during an echinococcosis screening programme in Kyrgyzstan. A total of 10 093 subjects were screened in the Osh, Naryn and Batken regions (oblast’, the first-level administrative region) of Kyrgyzstan (Fig. 1) in 2015–2017. Free screening was offered in arbitrarily selected city districts, towns and settlements. The screening schedule was made public *via* the system of general practice physicians, who informed the population in their districts, primarily *via* social networks. Only willing subjects were included. The number of unique study locations (settlements or streets for towns and cities) varied from 14 to over 100 for each district, and the number of subjects from each location varied from 1 to 147. A total number of unique study locations (city streets or settlements) was 1282. The number of settlements screened was about 464 (the number is approximate because the questionnaire had a city street or a settlement in the same field) out of a total of 795 settlements in the study regions. The number of subjects and settlements included in the study and total settlements in Batken, Naryn and Osh regions were 793/118/189, 477/34/137 and 8823/312/469, respectively. The screening included an abdominal ultrasound examination, ELISA for antibodies to CE (a commercial assay manufactured by Vector-Best, Novosibirsk, Russia) and AE and filling a risk questionnaire (Supplement 1).

### Statistics

The questionnaire included 38 questions regarding demographic information, potential risk factors, healthcare access and disease awareness. A number of questions presumed similar answers (e.g. ‘How many dogs do you have?’, ‘Do you have any contact with your dog?’, ‘Do your children play with the dog?’) or were meant to detail other questions (e.g. ‘How do you keep your dogs?’ or ‘Do you feed your dog livestock guts?’). Preliminary analysis suggested that answers to such questions were correlated. Owning certain kinds of livestock was also correlated. A few questions were related to AE risk factors (‘Does your dog chase mice?’, ‘Do you hunt predatory animals (fox, wolf)?’). Agricultural activities were excessively detailed (growing vegetables in fruit in general, for personal consumption and for sale) and were combined into ‘any agricultural activity’. Categorical variables (Residence area and Occupation) were converted to multiple binary indicator variables. Simple and multiple logistic regression was calculated in GraphPad Prism 8.4.3 (GraphPad Software LLC).

### Ethical statement

Ethical approval for the study was granted by the ethics committee of the Ministry of Health of Kyrgyzstan. Subjects diagnosed with echinococcosis were included in the state registry. As a rule, treatment with albendazole was conducted for cysts <3 cm (<1 cm in children) and surgical treatment for larger cysts. Positive subjects were being followed up at the submission date.

## Results

A total of 364 out of 10 093 study subjects were positive for CE ELISA. Of them, 329 had ultrasound findings compatible with CE, including liver CE (139), post-resection state (176) and other diagnoses compatible with CE, usually a liver cyst not unambiguously attributable to CE. Additional 124 post-resection patients were negative by ELISA; however, there were no patients with an active CE, but negative by ELISA. Only patients with ultrasound or surgical (post-resection) evidence of CE were included as positive (*n* = 453). The 35 ELISA-positive and ultrasound-negative subjects were excluded from the analysis. While they could include lung CE patients (and their number relative to the liver CE findings would indeed be compatible with the generally observed lung CE proportion between 1:6 and 1:12) (Larrieu and Frider, 2001), ELISA alone was not reliable to establish CE diagnosis due to a risk of false-positive results (Kronenberg *et al*., 2022). Also, 855 patients with ultrasound or ELISA evidence of AE were excluded (to be analysed in a separate study), leaving 8750 negative (control) subjects. The resulting sample included 9203 subjects, 2888 men and 6315 women. Post-resection patients were analysed together with the newly diagnosed patients.

There was no difference in CE prevalence relative to gender (odds ratio [OR] = 1.13 for men). The age distribution of CE prevalence was relatively even (Table 1), with somewhat higher prevalence at an age over 60 (OR = 1.33, *P* < 0.05 vs other age groups combined). This profile was compatible with the recent emergence of the epidemic, because elderly subjects did not accumulate exposure over their whole lives proportional to their age. Expectedly, the fraction of post-resection cases was somewhat lower among younger patients, 57% of the total prevalence in the age group <20 years old compared to 67% in other age groups; however, the difference was not significant (Table 1).

**Table 1.** CE prevalence (post-resection or positive by both serology and ultrasound) over age in south and central Kyrgyzstan

Age (years)	Number of study subjects	Total positive for CE	Number of post-resection patients (%)	Prevalence (%)
<20	1230	44	25 (56.8)	3.58
20–29	2858	104	80 (76.9)	3.64
30–39	2116	104	61 (58.7)	4.91
40–49	1519	77	52 (67.5)	5.07
50–59	1345	64	46 (71.9)	4.76
Over 60	1025	60	36 (60.0)	5.85
Total	10 093	453	300 (66.2)	4.49

Although the study covered 3 administrative regions of Kyrgyzstan, most subjects (8823, of them 8006 included in risk analysis) were from the Osh region. This allowed analysing prevalence and risk factors across distinct districts (raions, the second administrative division level) of the Osh region. The mean CE prevalence in the study was 4.5% and varied from 0.17 to 25.2% across the study regions (Table 2). All study regions were comparable in terms of farming habits: 70–99% of the subjects reported owning livestock and 43–92% – practicing non-organized agriculture. An apparent connection between the prevalence of CE and agriculture among study regions was not statistically significant. Most participants in all regions (96–100%) reported access to organized water supply, although the questionnaire did not specify which water source was used predominantly. Reported use of well water varied between 2.8 and 9.1% in all regions except for Nookat, which stood out at 34.7% (Table 2). Correlation between CE incidence and well water use was statistically significant, but not observed at all upon exclusion of a single outlier point (Nookat district).

**Table 2.** Prevalence of CE (post-resection or positive by both serology and ultrasound) and household practices in the study regions

Region (oblast’)	District	Number of study subjects	Positive for CE	CE prevalence (%)	Agriculture (%)	Own livestock (%)	Use of well water^a^ (%)
Batken	All	793	35	4.4	70	99	4.3
Naryn	All	477	52	10.9	77	90	6.3
Osh	Alay	1535	42	2.7	48	70	2.8
	Aravan	573	1	0.2	43	94	3.6
	Chong-Alay	1424	21	1.5	80	78	9.1
	Kara-Kulja	275	17	6.2	46	61	4.4
	Kara-Suu	1829	12	0.7	70	76	7.0
	Nookat	337	85	25.2	92	79	34.7
	Uzgen	2850	189	6.6	63	81	2.8
Total		10 093	453	4.5	66	78	5.9

a In addition to other water sources.

Uneven prevalence of CE across the study regions corresponded to a district being among the most prominent risk factors upon simple and multiple logistic regression analysis (Table 3). The only occupation significantly associated with a decreased CE risk upon simple (but not multiple) regression analysis was housework (OR = 0.76, *P* < 0.01). Teacher/child care occupation and retired status were associated with higher CE risk upon simple, but not multiple regression analysis. Other professions did not significantly affect the CE prevalence. Noteworthily, farming was associated with a weak negative risk (simple OR = 0.84), although this was not statistically significant.

**Table 3.** Risk factors of CE prevalence (post-resection or positive by both serology and ultrasound)

Risk factor	CE pos./neg. with risk	CE pos./neg. without risk	Simple OR	Simple OR 95% CI	Simple OR statistical significance	Multiple regression OR	Multiple regression OR 95% CI
Gender (male)	154/2735	300/6015	1.13	0.92–1.38	NS	1.04	0.80–1.35
Age 60+	60/901	392/7849	**1.47**	**1.01–1.94**	***P* = 0.01**	1.24	0.85–1.77
*Residence area*							
Batken region	35/752		0.96	0.66–1.35	NS	ND	ND
Naryn region	52/359		3.0	**2.19–4.05**	***P* < 0.0001**	**2**.**4**	**1.60–3.58**
Osh region							
Alay	42/758		1.07	0.76–1.46	NS	ND	ND
Aravan	1/571		0.06	**0.01–0.20**	***P* < 0.0001**	**0**.**09**	**0.02–0.30**
Chong-Alay	21/1377		0.26	**0.16–0.39**	***P* < 0.0001**	**0**.**33**	**0.19–0.53**
Kara-Kulja	12/1790		1.31	0.76–2.09	NS	ND	ND
Kara-Suu	17/251		**0**.**11**	**0.06–0.19**	***P* < 0.0001**	**0**.**15**	**0.08–0.26**
Nookat	85/246		**8**.**01**	**6.11–10.42**	***P* < 0.0001**	**6**.**10**	**4.08–9.12**
Uzgen	189/2646		**1**.**64**	**1.35–1.99**	***P* < 0.0001**	**1**.**58**	**1.19–2.12**
*Occupation*							
Farming	34/783		0.82	0.57–1.16	NS	ND	ND
Housework	138/3205		**0**.**76**	**0.62–0.93**	***P* < 0.01**	0.92	0.69–1.22
School pupil	30/691		0.83	0.55–1.18	NS	ND	ND
Teaching and child care	34/447		**1**.**50**	**1.03–2.13**	***P* < 0.05**	1.28	0.83–1.92
Medical	35/682		0.98	0.68–1.38	NS	ND	ND
Retired	49/473		**2**.**14**	**1.57–2.87**	***P* < 0.0001**	1.15	0.75–1.75
Unemployed	85/1833		0.87	0.68–1.10	NS	1.20	0.87–1.65
Dog ownership	297/5143	156/3607	**1**.**33**	**1.09–1.62**	***P* < 0.01**	1.09	0.86–1.40
Contact with a dog	185/3247	268/5500	1.17	0.96–1.42	NS	1.05	0.82–1.33
Cat ownership	122/1217	331/7533	**2**.**28**	**1.83–2.83**	***P* < 0.0001**	1.33	0.97–1.79
Contact with a cat	82/834	371/7916	**2**.**09**	**1.62–2.67**	***P* < 0.0001**	1.25	0.89–1.74
Hunting wild canids	17/118	436/8632	**2**.**87**	**1.64–4.64**	***P* < 0.001**	1.78	0.93–3.26
Sheep ownership	260/5116	193/3630	0.96	0.79–1.16	NS	ND	ND
Goat ownership	238/4240	215/4510	1.18	0.98–1.43	NS (*P* = 0.08)	**0**.**77**	**0.61–0.97**
Cow ownership	359/6161	94/2589	**1**.**61**	**1.28–2.04**	***P* < 0.001**	**1**.**34**	**1.01–1.80**
Horse ownership	259/3691	194/5059	**1**.**82**	**1.51–2.21**	***P* < 0.0001**	**1**.**50**	**1.18–1.91**
Any agricultural activity	377/7051	77/1699	1.18	0.92–1.53	NS	1.09	0.83–1.46
Any community water supply^a^	53/8448	85/302	**0**.**44**	**0.31–0.65**	***P* < 0.0001**	**0**.**56**	**0.38–0.84**
River^a^	159/3774	294/4976	**0**.**71**	**0.58–0.86**	***P* < 0.001**	**1**.**31**	**1.02–1.67**
Creek^a^	61/2444	392/6306	**0**.**40**	**0.30–0.52**	***P* < 0.0001**	**0**.**41**	**0.29–0.57**
Well^a^	51/515	402/8233	**2**.**02**	**1.48–2.72**	***P* < 0.0001**	0.92	0.62–1.35

Values with statistical support or confidence interval not including 1 are shown in bold. NS, not significant; ND, not conducted because simple OR *P* > 0.25; 95% CI, 95% confidence interval.

a Water source questions allowed multiple choice.

The risk questionnaire included demographics and potential risk factors. There was a certain discordance in answers to apparently redundant questions, such as dog (cat) ownership and acknowledged contact. Therefore, these factors were analysed separately.

Owning a dog or having contact with a dog was not associated with CE prevalence (Table 3). However, analysis of dog-specific risk factors among dog owners suggested that close contact (children playing with a dog) and feeding guts of slaughtered animals to dogs were associated with a significant risk of CE (Table 4), while regular disposing of dog feces (as opposed to leaving it on the ground) reduced the risk almost 2-fold with single test odd ratio (sOR) = 0.57 and multiple test odds ratio = 0.60). As there was a significant variation in terms of CE prevalence among the study regions, risk factor analysis was also conducted for distinct areas. Dog ownership was a statistically significant risk factor (OR = 2.67) only in the Nookat district (Table 5, only statistically significant findings are shown, *P* values are not corrected for multiple comparison); in all but 1 other district it could be suggested as a risk factor, but this was not statistically supported. Similarly, contact with a dog was a significant risk factor only in the Nookat district of the Osh region and in the Naryn region (Table 5). It is noteworthy that these were the areas with the highest CE prevalence (Table 2).

**Table 4.** Risk factors of CE prevalence among dog owners

Risk factor	Simple OR	Simple OR 95% CI	Simple OR statistical significance	Multiple test OR	Multiple test OR 95% CI
Contact with a dog	1.24	0.98–1.57	NS	0.94	0.67–1.32
Children play with a dog	**1**.**36**	**1.072**–**1.719**	***P* < 0.05**	1.38	0.99–1.93
Dog kept leashed	1.20	0.92–1.55	NS	0.96	0.72–1.27
Dog regularly dewormed	0.87	0.68–1.10	NS	0.91	0.70–1.17
Dog feces are disposed	**0**.**57**	**0.35**–**0.88**	***P* < 0.01**	**0.60**	**0.36–0.93**
Dog feeding with guts of slaughtered livestock	**1**.**52**	**1.18**–**1.94**	***P* < 0.01**	1.28	0.98–1.67
Cat ownership	**1**.**97**	**1.52**–**2.54**	***P* < 0.0001**	1.33	0.95–1.85
Contact with a cat	**2**.**17**	**1.62**–**2.87**	***P* < 0.0001**	**1**.**47**	**1.03**–**2.08**
Sheep ownership	0.80	0.63–1.02	NS	**0**.**73**	**0.54**–**0.97**
Goat ownership	1.03	0.82–1.31	NS	0.78	0.56–1.05
Cow ownership	**1**.**49**	**1.09**–**2.07**	***P* < 0.05**	1.42	0.99–2.07
Horse ownership	**1**.**78**	**1.40**–**2.27**	***P* < 0.0001**	**1**.**79**	**1.34**–**2.40**
Any agricultural activity	0.95	0.69–1.34	NS	0.99	0.70–1.41

Values with statistical support or confidence interval not including 1 are shown in bold. NS, not significant.

**Table 5.** Risk factors of CE prevalence with a significant statistical support within specific groups

Risk factor	Within a group	CE pos./neg. with risk	CE pos./neg. without risk	OR	OR 95% CI	Statistical significance
Contact with a dog	Age <20	23/298	21/646	2.37	1.29–4.36	*P* < 0.01
	Age 20–29	49/979	55/1589	1.45	0.98–2.14	*P* < 0.01
Dog ownership	**Nookat dist.**	65/135	20/111	2.67	1.55–4.78	*P* < 0.01
Contact with a dog	**Nookat dist.**	53/106	32/139	2.15	1.3–3.6	*P* < 0.01
	**Naryn reg.**	29/118	22/241	2.69	1.48–4.89	*P* < 0.01
Contact with a cat	Alay dist.	11/46	31/712	5.49	2.5–11.35	*P* < 0.0001
	**Kara-Kulja dist.**	3/11	14/240	4.68	0.97–17.13	*P* < 0.05
	**Uzgen dist.**	23/188	166/2458	1.81	1.14–2.87	*P* < 0.05
Contact with a cat	Dog owned	68/619	229/4524	2.17	1.62–2.87	*P* < 0.0001
Contact with a cat	Retired	18/50	35/458	4.7	2.45–8.85	*P* < 0.0001
Contact with a cat	Not retired	64/784	337/7458	2.09	1.62–2.67	*P* < 0.0001
Retired	Contact with a cat	18/50	64/784	4.41	2.38–7.89	*P* < 0.0001
Sheep ownership	**Kara-Kulja dist.**	15/100	2/151	11.33	3.10–72.8	*P* < 0.01
	**Nookat dist.**	48/92	37/154	2.17	1.32–3.6	*P* < 0.01
	**Uzgen dist.**	98/1761	91/883	0.54	0.4–0.73	*P* < 0.0001
Goat ownership	**Kara-Kulja dist.**	11/58	6/193	6.10	2.22–18.38	*P* < 0.001
	**Nookat dist.**	76/152	9/94	5.22	2.5–11.63	*P* < 0.001
	**Uzgen dist.**	76/1439	113/1207	0.56	0.42–0.76	*P* < 0.001
Cow ownership	**Kara-Kulja dist.**	15/119	2/132	8.32	2.28–53.47	*P* < 0.001
	**Nookat dist.**	78/172	7/74	4.79	2.25–11.87	*P* < 0.001
	**Uzgen dist.**	154/1944	35/702	1.59	1.10–2.30	*P* < 0.01
Horse ownership	**Kara-Kulja dist.**	14/42	3/209	23.22	7.20–104	*P* < 0.0001
	**Nookat dist.**	66/110	19/136	4.3	2.47–7.76	*P* < 0.0001
	**Uzgen dist.**	105/1346	84/1300	1.21	0.9–1.62	NS
Gardening for own consumption	Alay dist.	25/328	17/430	1.93	1.03–3.69	*P* < 0.05
	**Kara-Kulja dist.**	16/118	1/133	18.03	3.6–328	*P* < 0.01
	**Naryn region**	35/301	16/58	0.42	0.22–0.81	*P* < 0.01
	**Nookat dist.**	51/192	34/54	0.42	0.25–0.72	*P* < 0.01
	**Uzgen dist.**	132/2052	57/594	0.67	0.49–0.93	*P* < 0.05
Hunting	**Nookat dist.**	12/9	73/237	4.33	1.76–11	*P* < 0.01
Hunting	Retired	7/9	46/499	8.44	2.90–23.7	*P* < 0.0001
Retired	Hunters	7/9	10/109	8.48	2.6–28.11	*P* < 0.001
Retired	Cat ownership	26/80	96/1137	3.85	2.33–96.21	*P* < 0.0001
Retired	Contact with a cat	18/50	64/784	4.41	2.38–7.89	*P* < 0.0001
Cat ownership	Retired	26/80	27/428	5.15	2.85–9.31	*P* < 0.0001
Contact with a cat	Retired	18/50	35/458	4.7	2.45–8.85	*P* < 0.0001
Using well water	Access to any organized or running water	50/497	378/8112	2.16	1.59–2.94	*P* < 0.0001

NS, not significant.

Areas with CE prevalence over 5% are shown in bold.

Owning small livestock (sheep and goats) was not associated with CE in the overall sample, whereas cows and, especially, horses were associated with elevated CE risk upon both simple and multiple regression analyses (Table 3). The same pattern and similar ORs for owning livestock (no risk for goats and sheep, risk for cows and horses) were observed among dog owners (Table 4), thus there was no evidence that dogs and livestock had a synergetic effect on CE prevalence.

Sheep and goats were significant risk factors in 2 highly endemic regions, Kara-Kulja and Nookat, with simple ORs between 2.17 and 11.3 (Table 5). However, the risks associated with a particular kind of livestock were not consistent among districts. In the Uzgen district (also highly endemic), both sheep and goats were associated with a significant negative risk (OR = 0.54 and 0.56, respectively), whereas cows and horses were positively and significantly associated with CE risk in all these highly endemic regions (Kara-Kulja, Nookat and Uzgen). It is also noteworthy that in some districts, CE prevalence was low despite universal livestock ownership (Aravan district, 94% own at least 1 kind of livestock, 0.2% CE prevalence).

Agriculture (growing fruits and vegetables) was not a significant risk in the overall sample (Table 3). It could be either a positive or a negative risk factor in distinct districts (Table 5), but with relatively high *P* values even in the absence of multiple comparison correction.

Cats (both ownership and contact) were a significant risk factor for CE prevalence with ORs consistently higher than dog ownership and contact (Table 3). Cats are not generally known as CE risk factor, thus to exclude a statistical artefact, the effect was analysed further. In all but 1 study regions, cats were indeed a risk factor, but this was statistically supported only in 3 regions (Table 5). Noteworthily, these regions were distinct from those with dogs as a significant risk factor. The OR values of contact with a cat in the overall sample (Table 3) and in distinct districts were much higher than those from contact with a dog. Contact with a cat could be potentially associated with a lifestyle typical for an elderly age or retirement. However, contact with a cat was a significant additional risk factor in both non-retired and retired occupation groups when analysed separately (Table 5). Another plausible explanation could be that cats physically facilitate CE transfer from dogs to humans. However, an OR for dog owners from contact with a cat (2.17) was the same as for the general sample (2.09) (Table 5).

Organized water supply or using running water from a river or a creek were significant negative risk factors (OR = 0.4–0.71) of CE upon simple regression analysis. This was supported by multiple regression analysis for tap water and a creek, but not for river water, which is likely a statistical oddity (Table 3). Meanwhile, use of wells was a significant positive risk factor for CE (sOR = 2.03), but not upon multiple regression analysis. It is likely that much of the impact of this factor came from Nookat district, which stands out for its very high prevalence of well usage and the highest CE prevalence (Table 2). This risk added up with livestock ownership (OR = 2.92, *P* < 0.0001 when 2 factors were present) and was further amplified by dog ownership (OR = 3.88, *P* < 0.0001 when 3 factors were present). The role of the combination of these 3 risks was the strongest in the hyperendemic Nookat district (OR = 11.65, *P* = 0.001). Among subjects with this combination of risk factors (Nookat + well + dog + livestock), 37 of 39 (95%) had CE. While this single observation should not be generalized, it highlights the high unevenness of CE risk factors in Kyrgyzstan.

## Discussion

Kyrgyzstan is known for a high incidence and prevalence of both CE and AE. In our data, the overall CE prevalence in endemic regions was 4.5%. This is close to an estimate of 3.4% published almost 20 years ago (Torgerson *et al*., 2003) and also compatible with a surgical incidence of up to 176 per 100 000 per year in the most affected districts (Paternoster *et al*., 2020). The CE prevalence varied more than 100-fold between the study regions, which is also concordant with reports of highly variable regional incidence data (Paternoster *et al*., 2020).

The occupation status significantly increasing CE prevalence was being retired. This corresponded to higher CE prevalence among the 60+ age group. Previously, an almost 2-fold higher incidence among the retired was reported in Kara-Suu district of the Osh region (Kholmatova and Grjibovski, 2016), and age has been associated with an increased CE risk in several studies (reviewed in Wang *et al*., 2014). This could be potentially explained by cumulative exposure over life. However, such a prevalence cumulation trend was generally not observed across all age groups, and the fraction of post-resection patients did not increase significantly with age. Retired and housework occupations were risk factors of CE in Turkey (Tamarozzi *et al*., 2019). In 2003 in neighbouring Kazakhstan, children and people with occupations associated with animal husbandry were at the highest risk of infection (Shaikenov *et al*., 2003). In this study, farming was not associated with CE risk, whereas housework was a single occupation associated with a decreased prevalence of CE.

Classical CE risk factors, such as livestock and contact with a dog, were only moderately associated with CE prevalence in the overall sample (OR below 2). However, this association was significantly stronger in the highly endemic districts. Most noteworthy, dogs, the definitive host and usually the main source of CE infection, were not the highest risk factor, with OR between 2.17 and 2.69 even in the hyper-endemic districts. This was unexpected, because about 10% of dogs in endemic districts were infected with *E. granulosus* upon autopsy (Raimkulov *et al*., 2018). Another study reported a 19% prevalence of *E. granulosus* in dogs in the Naryn region in 2005 (Ziadinov *et al*., 2008). In 2003 in neighbouring Kazakhstan, 5.8% of village dogs and 23.2% of shepherd dogs were infected with *E. granulosus* (Shaikenov *et al*., 2003). In other countries, the risk associated with dogs was highly variable, from almost none in Jordan (Dowling *et al*., 2000) and Mongolia (Dorjsuren *et al*., 2020) to moderate in Argentina (Larrieu *et al*., 2002) and up to OR > 3 in a meta-review of several reports (Possenti *et al*., 2016). One explanation may be that dogs pose not just personal, but also community risk, thus even those not reporting this risk factor were actually exposed. This is possible because the eggs can spread over 100 m from the feces deposition site (Sanchez Thevenet *et al*., 2019), and in 1 study, 5 of 120 soil samples in gardens were positive (Shaikenov *et al*., 2004). This hypothesis of indirect exposure is in line with a recent discussion on the importance of soil as a source of CE (Tamarozzi *et al*., 2020) and concordant with the role of water supply-related risk factors. Indeed, access to purified or running water was a strong and significant factor decreasing CE risk almost 2-fold, which is concordant with reports from Argentina (Larrieu *et al*., 2002). On the contrary, use of well water was significantly associated with a higher CE risk (sOR = 2.03). It is noteworthy that the association of CE with a poor water supply has also been implicated as a CE risk factor in other studies (Torgerson *et al*., 2009*a*). Contaminated wells have been implicated as a potential infection source also in Africa (Macpherson, 2001). It remains to be observed if contaminated water was a direct infection source, or whether the limited water supply led to sparse use of water for sanitary purposes, and thus poor sanitation.

Small livestock (sheep and goats) were a strong risk factor only in the highly endemic Nookat and Kara-Kulja districts (sOR between 2.17 and 11.33), but not in other regions with a comparably high CE prevalence, the Naryn region and the Uzgen district of the Osh region (OR between 1.09 and 1.59). Livestock in Central Asia is ubiquitously infected with CE. One study reported that 64% of sheep in Kyrgyzstan had cysts in 2006 (Torgerson *et al*., 2009*b*). Another study indicated a CE prevalence of 51.2% in small livestock and 11.2% in cows (Kholmatova and Grjibovski, 2016). It is possible that livestock, just like dogs, posed a community rather than personal risk, which made it difficult to gain statistical support for a personal risk. Overall, the typical dog–livestock risk pattern was fully supported only in the highly endemic (25% prevalence) Nookat district (and, to lesser extent, in another highly endemic district, Kara-Kulja), and coincides with an appalling adherence to dog deworming (8.4%). On the contrary, Alay district stood out by both good adherence to deworming (55%) and a complete absence of risk associated with dogs (OR = 1.02) and livestock (OR = 0.98). This may explain the moderate prevalence of CE (2.7%) despite almost universal livestock ownership (69.5%) and common ownership of both dogs and livestock (28% compared to 34% in the hyperendemic Nookat district). Interestingly, deworming adherence was not a significant personal risk factor in this study and also in Mongolia (Dorjsuren *et al*., 2020), supporting its importance as a community measure. A careful disposal of dog feces, on the contrary, was a strong negative risk factor, again compatible with indirect, rather than direct, transmission of the eggs.

Cats, rather than dogs, were a strong risk factor in the overall sample, but especially in a low-prevalence Alay district (OR = 5.49). This was not likely to be a statistical oddity, because both ownership and contact with cats were implicated as moderate, but statistically significant risk factors across other study regions. There was no obvious association of cat ownership with age and occupation (e.g. a ‘retired lifestyle’). Cats are known as definitive hosts of *E. multilocularis* (Petavy *et al*., 2000). There are no reports of *E. granulosus* infecting them as definitive hosts; moreover, cats may be infected by the larval stage of *E. granulosus* as intermediate hosts (Konyaev *et al*., 2012; Bonelli *et al*., 2018). Thus, their ‘mechanical’ role as egg vectors remains the only explanation.

A dissimilar role of conventional and less common risk factors in distinct regions of Kyrgyzstan implies that the high overall CE prevalence in the country is not a holistic epidemic, but there are specific epidemiological conditions at least in different districts (and, maybe, in even smaller administrative units). This is compatible with the highly variable prevalence observed here and a reported uneven incidence of CE across the country (Paternoster *et al*., 2020). Several distinct risk profile patterns can be highlighted.

In 2 districts, Kara-Kulja and Nookat, there was a high prevalence of CE and a full set of classical risk factors (livestock–dog), and a low overall level of deworming adherence (15.7 and 8.4% of dog owners, respectively). In other highly endemic areas, the Naryn region and the Uzgen district of the Osh region, there was no statistically supported evidence of livestock and dogs as individual risk factors. High deworming adherence in these areas (57 and 51%, respectively) could diminish personal risks. In Alay district, there was a relatively low CE prevalence, high deworming adherence (55%) and lack of risk from livestock–dog-related factors. Most likely, here efficient prevention has largely broken the transmission cycle. Lower effect of conventional risk factors (and, probably, higher public awareness in this regard) in Alay district could explain why unconventional factors, such as contact with a cat, came out as the key risk factors.

A detailed analysis of CE risk factors is hampered by the weaknesses of the dataset used in this study. The screening was conducted primarily to identify patients with CE and AE, while risk analysis was a secondary result. Voluntary participation led to a biased dataset, as exemplified by the male:female ratio of 31:69. Consequently, it is possible that certain occupations, such as pastoral farming, were underrepresented. Sampling efficiency could also be biased by local general practitioners voluntarily inviting certain groups more persistently than others. For example, 38% of female subjects aged 21–30 were pregnant by ultrasound examination, compatible with this suggestion. These considerations are partially offset by many study locations (over 1200) and a large dataset. Also, findings that were paralleled in distinct districts (such as cats as a significant risk factor) are less prone to sample bias resulting, for example, from family CE foci. Incidence difference between districts, which could be more than 100-fold, is another weakness of the dataset. Even though risk factor analysis was conducted for distinct districts, there is no reason to believe that CE endemicity corresponds to administrative borders, thus similar variations of incidence could not be excluded at a lower administrative level.

The questionnaire also had limitations. Answers to many partially redundant questions were correlated, which could complicate multiple regression analysis or a reasonable multiple comparison correction. Although the results of single and multiple regression analysis generally correlated well, the limited suitability of the dataset for multiple regression analysis can be exemplified by a risk from river water access, which was the opposite in 2 kinds of the analysis. Discrepancy between such risk factors as dog/cat ownership and contact highlight potential communication issues. A significant risk associated with hunting wild canids (included in the questionnaire as a potential AE risk factor) exemplifies statistical oddities, likely affecting CE prevalence *via* unclear lifestyle associations. Therefore, findings should be interpreted with care and should be considered when organizing a national screening in Kyrgyzstan that might yield more reliable results.

Despite many studies, there is currently no comprehensive understanding of the CE infection routes, and conflicting or inconclusive reports are common (Eckert and Deplazes, 2004), even among studies conducted in the same region (Wang *et al*., 2014). In line with the global pattern, the CE epidemic in Kyrgyzstan is not holistic in terms of potential mechanisms and risk factors. High variability of incidence between districts corresponds to different risk profiles. Thus, certain areas can greatly benefit from preventive measures that will have limited efficiency elsewhere. There is no guarantee that the variability of risk patterns has been explored exhaustively here; however, the high incidence of CE in Kyrgyzstan allows gaining statistical power even for small samples and marginal risk factors. Further studies should go to even lower administrative units, because risk factors and the corresponding mitigation measures may differ at administrative levels below a district.

## Supporting information

Raimkulov et al. supplementary material Raimkulov et al. supplementary material

## Data Availability

Data available on request due to privacy/ethical restrictions.

## Supplementary material

The supplementary material for this article can be found at https://doi.org/10.1017/S0031182024000763.
