# Supplementary material for: Dissimilar local risk factors among patients diagnosed with cystic echinococcosis upon voluntary screening in highly endemic regions of Kyrgyz Republic
Source: Parasitology. 2024 Dec 3;151(10):1118–25. doi: 10.1017/S0031182024000763 (PMC11894009; doi:10.1017/S0031182024000763)
Supplement: Raimkulov et al. supplementary material [file S0031182024000763sup001.docx]

**Map (questionnaire) of the population survey on the presence of risk factors for parasitic diseases invasion**

**Passport details**:

**№ ________­­­­_________ Date of filling: "____" _______­­­­______ 20_____**

| **1.** | Family name__________________, First name _____________________________ | | | | | | | | | | | | | | | | | | | | |
| --- | --- | --- | --- | --- | --- | --- | --- | --- | --- | --- | --- | --- | --- | --- | --- | --- | --- | --- | --- | --- | --- |
| **2.** | Age: ____ *years* | | **3.** | | Nationality: ___________ | | | | | | | | | | | | **4.** | Gender: *male/female* | | | |
| **5.** | **Address:** ____________________ region, ______________________district,  village __________________________________, street_____________________ | | | | | | | | | | | | | | | | | | | | |
| **6.** | Profession: ___________________ | | | | | | | | | **7.** | | | Monthly income: _________ *som* | | | | | | | | |
| **Risk factors**: | | | | | | | | | | | | | | | | | | | | | |
| **8.** | Do you know about the disease of echinococcosis/alveococcosis? *Yes No* | | | | | | | | | | | | | | | | | | | | |
| **9.** | Have you been examined by ultrasound in recent years? *Yes No* | | | | | | | | | | | | | | | | | | | | |
| **10.** | How many dogs do you have? | | | | | | | | ___________ | | | | | | | | | | | | |
| **11.** | How do you keep your dogs? | | | | | | | | a) *Always roaming free*, b) *Tied* | | | | | | | | | | | | |
| **12.** | Have dogs been dewormed? | | | | | | | | | | *Yes No*  If yes, how many times a year _________ | | | | | | | | | | |
| **13.** | Do you have contact with your dog? | | | | | | | | | | *Yes No* | | | | | | | | | | |
| **14.** | Do your children play with the dog? | | | | | | | | | | *Yes No* | | | | | | | | | | |
| **15.** | What do you do with your dogs' feces? | | | | | | | | | | | | | ___________________________ | | | | | | | |
| **16.** | Does your dog chase mice? | | | | | | *Yes No* | | | | **17.** | | | Do you have a cat? | | | | | *Yes No* | | |
| **18.** | Do you have contact with a cat? | | | | | | | | | | *Yes No* | | | | | | | | | | |
| **19.** | Do you feed livestock guts to your dog? | | | | | | | | | | | | | | | | | | *Yes No* | | |
| **20.** | Do you hunt predatory animals (fox, wolf)? | | | | | | | | | | | | | | | | | | *Yes No* | | |
| **21.** | Have you ever had echinococcosis or alveococcosis? | | | | | | | | | | | | | | | | | | *Yes No* | | |
| **22.** | Do you have sheep? *Yes No* | | | | | | | | | | | **23.** If yes, how many _ _ _ _ _ (enter) | | | | | | | | |  |
| **24.** | Do you have goats? *Yes No* | | | | | | | | | | | **25.** If yes, how many _ _ _ _ _ (enter) | | | | | | | | |  |
| **26.** | Do you have cows? *Yes No* | | | | | | | | | | | **27.** If yes, how many _ _ _ _ _ (enter) | | | | | | | | |  |
| **28.** | Do you have horses? *Yes No* | | | | | | | | | | | **29.** If yes, how many _ _ _ _ _ (enter) | | | | | | | | |  |
| **30.** | Do you grow vegetables and fruits? | | | | | | | | | | | | | | | | | | *Yes No* | |  |
| **31.** | Does the dog and cat have access to the garden? | | | | | | | | | | | | | | | | | | *Yes No* | |  |
| **32.** | Do you grow vegetables and fruits for sale? | | | | | | | | | | | | | | | | | | *Yes No* | |  |
| **33.** | Do you grow vegetables and fruits for your personal use? | | | | | | | | | | | | | | | | | | *Yes No* | |  |
| **34.** | Do you use water  from a central water supply? | | | | Tap in the house | | | | | | *Yes No* | | | | Tap on the street | | | | | *Yes No* |  |
|  |  |  |  |  | River | | | | | | *Yes No* | | | | Pump in the yard | | | | | *Yes No* |  |
|  |  |  |  |  | Stream | | | | | | *Yes No* | | | | Well | | | | | *Yes No* |  |
| **Diagnostics:** | | | | | | | | | | | | | | | | | | | | |  |
| **35.** | Ultrasound ___ _ _ _ | 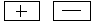 | | | | **36.** | | What organ is the cyst found in?:___________ | | | | | | | | | | | | |  |
| **37.** | Type of cyst ______ | | | 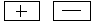 ____(fill in) | | | | | | | | | | **38.** | | ELISA _ _ _ _ _ | | | | 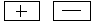 |  |

**Signature: ______________________ Phone number: _________________________**

**Карта (анкета) опроса населения о наличии факторов риска инвазии паразитарными болезнями**

**Паспортные данные**:

**№ ________­­­­_________ Дата заполнения: «____» _______­­­­______ 20_____ г.**

| **1.** | Ф _____________________________, Имя _____________________________ | | | | | | | | | | | | | | | | | | | | | |
| --- | --- | --- | --- | --- | --- | --- | --- | --- | --- | --- | --- | --- | --- | --- | --- | --- | --- | --- | --- | --- | --- | --- |
| **2.** | Возраст: ____ *лет* | | **3.** | | Национальность: ___________ | | | | | | | | | | | | **4.** | Пол: *муж / жен* | | | | |
| **5.** | **Адрес:** ____________________ область, ______________________район,  село __________________________________, ул. ______________________ | | | | | | | | | | | | | | | | | | | | | |
| **6.** | Профессия: ___________________ | | | | | | | | | **7.** | | | Месячный доход: _________ *сом* | | | | | | | | | |
| **Факторы риска**: | | | | | | | | | | | | | | | | | | | | | | |
| **8.** | Вы знаете, о болезни эхинококкоза/альвеококкоза? *Да Нет* | | | | | | | | | | | | | | | | | | | | | |
| **9.** | Вы последние годы обследовались ли на УЗИ? *Да Нет* | | | | | | | | | | | | | | | | | | | | | |
| **10.** | Сколько у вас собак (вписать)? | | | | | | | | ___________ | | | | | | | | | | | | | |
| **11.** | Как содержите Ваших собак? | | | | | | | | а) *Всегда бродит свободный*, б) *Привязан* | | | | | | | | | | | | | |
| **12.** | Проводили ли дегельминтизации собак? | | | | | | | | | | *Да. Нет*  Если да, сколько раз в год _________ | | | | | | | | | | | |
| **13.** | Имеете ли Вы контакт с собакой? | | | | | | | | | | *Да Нет* | | | | | | | | | | | |
| **14.** | Играют ли Ваши дети с собакой? | | | | | | | | | | *Да Нет* | | | | | | | | | | | |
| **15.** | Что Вы делаете с фекалиями Ваших собак? | | | | | | | | | | | | | ___________________________ | | | | | | | | |
| **16.** | Мышкует ли Ваша собака? | | | | | | *Да Нет* | | | | **17.** | | | Имеете кошку? | | | | | | *Да Нет* | | |
| **18.** | Имеете ли Вы контакт с кошкой? | | | | | | | | | | *Да Нет* | | | | | | | | | | | |
| **19.** | Даете ли вы собаке внутренности домашних животных? | | | | | | | | | | | | | | | | | | | *Да. Нет* | | |
| **20.** | Охотитесь ли Вы на хищных животных (лиса, волк)? | | | | | | | | | | | | | | | | | | | *Да. Нет* | | |
| **21.** | Болели ли Вы эхинококкозом или альвеококкозом? | | | | | | | | | | | | | | | | | | | *Да Нет* | | |
| **22.** | Имеете ли Вы овец? *Да Нет* | | | | | | | | | | | **23.** Если да, сколько _____ (вписать) | | | | | | | | | |  |
| **24.** | Имеете ли Вы коз? *Да Нет* | | | | | | | | | | | **25.** Если да, сколько _____ (вписать) | | | | | | | | | |  |
| **26.** | Имеете ли Вы коров? *Да Нет* | | | | | | | | | | | **27.** Если да, сколько _____ (вписать) | | | | | | | | | |  |
| **28.** | Имеете ли Вы лошадей? *Да Нет* | | | | | | | | | | | **29.** Если да, сколько _____ (вписать) | | | | | | | | | |  |
| **30.** | Выращиваете овощи и фрукты? | | | | | | | | | | | | | | | | | | *Да Нет* | | |  |
| **31.** | Имеет ли собака и кошка доступ к огороду? | | | | | | | | | | | | | | | | | | *Да Нет* | | |  |
| **32.** | Выращиваете овощи и фрукты для продажи? | | | | | | | | | | | | | | | | | | *Да Нет* | | |  |
| **33.** | Выращиваете овощи и фрукты для личного пользования? | | | | | | | | | | | | | | | | | | *Да Нет* | | |  |
| **34.** | Вы употребляете воду  центральное водоснабжение? | | | | Кран в доме | | | | | | *Да Нет* | | | | Кран на улице | | | | | | *Да Нет* |  |
|  |  |  |  |  | Речка | | | | | | *Да Нет* | | | | Колонка во дворе | | | | | | *Да Нет* |  |
|  |  |  |  |  | Ручей | | | | | | *Да Нет* | | | | Колодец | | | | | | *Да Нет* |  |
| **Диагностика:** | | | | | | | | | | | | | | | | | | | | | |  |
| **35.** | УЗИ ______ | 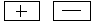 | | | | **36.** | | В каком органе обнаружена киста:___________ | | | | | | | | | | | | | |  |
| **37.** | Тип кисты _______ | | | 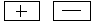 ____(вписать) | | | | | | | | | | **38.** | | ИФА ________ | | | | | 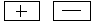 |  |

**Подпись: ______________________ Телефон: _________________________**
